# Protective Smell of Hydrogen Sulfide and Polysulfide in Cisplatin-Induced Nephrotoxicity

**DOI:** 10.3390/ijms20020313

**Published:** 2019-01-14

**Authors:** Xu Cao, Wencan Zhang, Philip K. Moore, Jinsong Bian

**Affiliations:** 1 Department of Pharmacology, Yong Loo Lin School of Medicine, National University of Singapore, Singapore 117600, Singapore; dprmpk@nus.edu.sg; 2 Food Science and Technology Program, Department of Chemistry, National University of Singapore, Singapore 117600, Singapore; a0134754@u.nus.edu; 3 National University of Singapore (Suzhou) Research Institute, Suzhou 215123, China

**Keywords:** hydrogen sulfide, hydrogen polysulfide, cisplatin-induced nephrotoxicity, NADPH oxidase, GYY4137, cystathionine γ-lyase

## Abstract

Though historically known as a toxic gas, hydrogen sulfide (H_2_S) has displayed a new face as the third endogenous gaseous signaling molecule after nitric oxide (NO) and carbon monoxide (CO). Here in this review, we survey the role and therapeutic potential of H_2_S in cisplatin-induced nephrotoxicity. Specifically, reduction of H_2_S by cystathionine γ-lyase (CSE) downregulation upon cisplatin treatment may contribute to cisplatin-induced renal cell injury, possibly by augmentation of endogenous reactive oxygen species (ROS) production, while H_2_S donation may prevent subsequent renal dysfunction by inhibiting NADPH oxidase activation. Intriguingly, H_2_S slow-releasing compound GYY4137 seems to increase the anticancer activity of cisplatin, at least in several cancer cell lines, and this is probably due to its own anticancer effect. However, the efficacy of H_2_S donors in tumor-bearing animals remains to be tested in terms of renal protection and cancer inhibition after receiving cisplatin. Furthermore, accumulative evidence regarding usage of polysulfide, a novel H_2_S derived molecule, in the therapy of cisplatin-induced nephrotoxicity, was also summarized.

## 1. Introduction

Hydrogen sulfide (H_2_S) was historically known as a toxic gas with a rotten-egg smell [1]; however, extensive studies in the last two decades have unveiled its far-reaching effects in mammalian physiology and resulted in its recognition as the third endogenous gaseous signaling molecule after nitric oxide (NO) and carbon monoxide (CO) [2,3,4].

In the renal system, the abundance of H_2_S is clearly evidenced by the presence of all four biosynthesizing pathways, including cystathionine γ-lyase (CSE), cystathionine β-synthase (CBS), 3-mercaptopyruvate sulfurtransferase (3-MST) coupled with cysteine aminotransferase (CAT), and 3-MST coupled with d-amino acid oxidase (DAO). Not surprisingly, compelling data has suggested the modulatory effect of H_2_S in renal physiology and its protection in several kidney diseases [3,5,6]. Interestingly, emerging studies in recent years have indicated the possible involvement of H_2_S in cisplatin-induced nephrotoxicity [7,8]. In this review, we aim to survey the potential roles and protective mechanisms of H_2_S in cisplatin-induced nephrotoxicity. Additionally, progress in the use of polysulfide, an H_2_S derived endogenous molecule, for the disease is also reviewed.

## 2. Biosynthesis of H_2_S and Hydrogen Polysulfides in the Kidney

### 2.1. Biosynthesis of H_2_S in the Kidney

**Figure 1:**
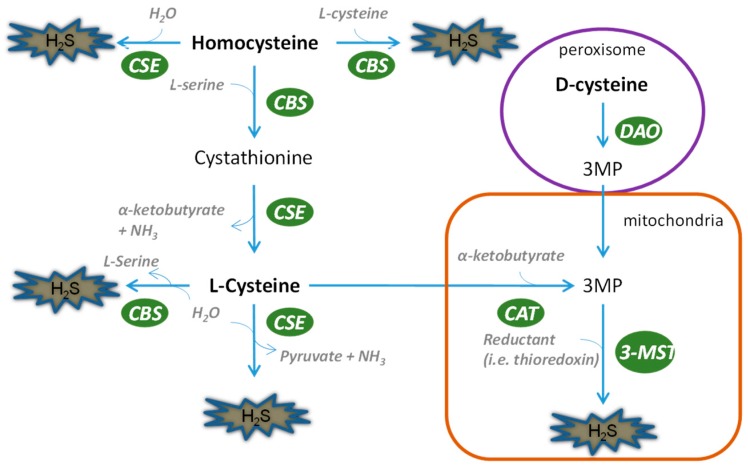
Biosynthesis of H_2_S in the kidney. The biological production of H_2_S in the kidney is mediated by four pathways. CSE and CBS can use l-cysteine and homocysteine as substrates to generate H_2_S in cytosol, while they can translocate into mitochondria in hypoxic states. l-cysteine and d-cysteine have to be catalyzed into 3MP before they can be utilized by 3-MST for the production of H_2_S. 3-MST mediates H_2_S production, mainly in mitochondria.

The kidney possesses all four of the H_2_S biosynthesis pathways (Figure 1), indicating the abundance of this gaseous molecule in the organ. Briefly, CSE leads to the generation of H_2_S by a two-step reaction, first dimerizing l-cysteine into thiocytsteine and then using it to react with other thiols to produce H_2_S. CBS can directly catalyze l-cysteine and homocystenin into H_2_S along with cystathinine. In contrast, 3-mercaptopyruvate has to be generated by CAT, after which 3-MST can use it as a substrate to produce pyruvate and H_2_S. Unlike CSE and CBS, 3-MST is mainly located in mitochondria and therefore regulates mitochondrial homeostasis of H_2_S. A fourth H_2_S generation pathway was recently discovered by Kimura’s group, in which d-cysteine serves as a substrate to produce H_2_S by d-amino acid oxidase coupled with 3-MST. For a detailed portrayal of H_2_S biosynthesis, it is advisable to refer to several excellent reviews published elsewhere [9,10,11]. It is worth mentioning that compared with its counterparts, the role of 3-MST is currently not well appreciated in either kidney physiology or disease in spite of its special role in producing H_2_S in mitochondria. This is an intriguing area warranting further exploration.

### 2.2. Biosynthesis of Hydrogen Polysulfide in the Kidney

**Figure 2:**
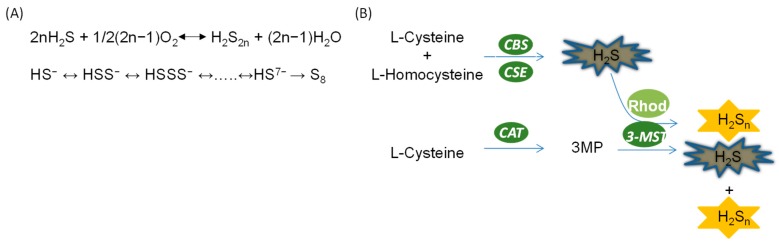
Generation of hydrogen polysulfides in the kidney. (**A**) Non-enzymatic production of hydrogen polysulfide; (**B**) enzymatic production of hydrogen polysulfide.

It is suggested that, similar to H_2_S, polysulfide is produced in mammals through both non-enzymatic pathways and enzymatic pathways (Figure 2). However, the proportion of endogenous polysulfide generated by these pathways remains elusive. Additionally, the regulation of polysulfide production is not well understood.

#### 2.2.1. Non-Enzymatic Generation of Polysulfide

In the presence of oxygen, polysulfide is generated from H_2_S as described in the following equation: 2n H_2_S + 1/2 (2n−1) O_2_ = H_2_S_2n_ + (2n−1) H_2_S [12]. Nevertheless, the reaction shown here with O_2_ is very slow unless it is catalyzed by a transition metal, and this may not occur much inside the cell. Moreover, HS^−^ is also able to react with sulfur, S^0^, to generate polysulfide containing various numbers of sulfurs [13]. When the number of sulfurs reaches eight, the sulfur molecule cyclizes and becomes stable. Recently, another reaction was identified in which polysulfide serves as a product of the interaction between H_2_S and NO [14]. Importantly, these reactions should be partially responsible for the production of polysulfide in mammalian systems as they can occur in physiological conditions [14].

#### 2.2.2. Enzymatic Generation of Polysulfide

Intriguingly, Kimura and others [15] found that hydrogen polysulfide, H_2_S_n_, can also be produced by an enzymatic reaction catalyzed by H_2_S-producing enzyme 3-MST. In the study, it was found that 3MP promoted the production of H_2_S_n_ in brain cells of wide-type mice but not 3-MST-deficient mice, suggesting that H_2_S_n_ may be exclusively produced by 3-MST. Moreover, purified recombinant 3-MST also facilitates the production of H_2_S_n_ in the presence of 3MP. However, mutation of the cysteine residue at the catalytic site of 3-MST leads to failure in the production of H_2_S and H_2_S_n_. Additionally, H_2_S can be used as a direct substrate for the production of H_2_S_n_ by 3-MST in the cooperation of rhodanese [15]. It is believed that polysulfide can also be generated by these pathways in other mammalian systems due to the universal presence of 3-MST [16].

## 3. Cisplatin-Induced Nephrotoxicity

Cisplatin is a simple compound, containing only a central platinum atom surrounded by two chlorine atoms and two ammonia groups in a cis configuration. Nevertheless, it is an extremely effective chemotherapeutic drug for cancer treatment. For example, it is widely used for the treatment of many solid tumors from areas such as head, neck, lung, testis, ovary, and breast [17]. However, its usage in cancer therapy is largely compromised due to its numerous adverse effects, which include nephrotoxicity, hearing loss, neurotoxicity, severe nausea, and myelosuppression [18]. Among these adverse effects, nephrotoxicity is the most prevalent; evidence shows that over 30% of patients show symptoms of acute kidney injury following the administration of cisplatin [19].

### 3.1. Clinical Features of Cisplatin-Induced Nephrotoxicity

Cisplatin-induced nephrotoxicity was initially described in clinical trials of cisplatin for cancer therapy [20], followed by similar observations in a number of species such as mice, rats, and dogs [21]. Typically, renal insufficiency manifests as increases in serum creatinine and blood urea nitrogen levels several days after the administration of cisplatin, along with a reduction of serum magnesium and potassium levels [22]. Meanwhile, urine output is normally preserved, and it usually contains glucose and small amounts of protein, indicating dysfunction of the proximal tubule [22]. Recovery of renal function usually occurs over a period of 2–4 weeks, provided cisplatin administration is discontinued; however, progressive and permanent nephrotoxicity may appear even with preventive measures [23,24].

### 3.2. Risk Factors of Cisplatin-Induced Nephrotoxicity

Several risk factors associated with cisplatin-induced nephrotoxicity have been identified. Accumulative evidence suggests that cumulative and/or high doses of cisplatin increase the rate of nephrotoxicity [25,26]. Besides this, other factors, such as smoking, older age, female sex, and pre-existing renal dysfunction have also been found to be associated with increased incidence of nephrotoxicity [27,28]. However, whether pre-existence of chronic kidney disease influences the occurrence of cisplatin nephrotoxicity remains elusive, due to limited data reported. In contrast, diabetes was reported to lower the risk of cisplatin-induced nephrotoxicity in rats [29]; however, this effect was not observed in human clinical trials [30,31]. Moreover, certain mutations in the gene of organic cation transporter 2 (OCT2) may be associated with a lower risk of nephrotoxicity, probably because OCT2 regulates the transportation of platinum into kidney cells [32,33].

### 3.3. Disease Pathophysiology of Cisplatin-Induced Nephrotoxicity

**Figure 3:**
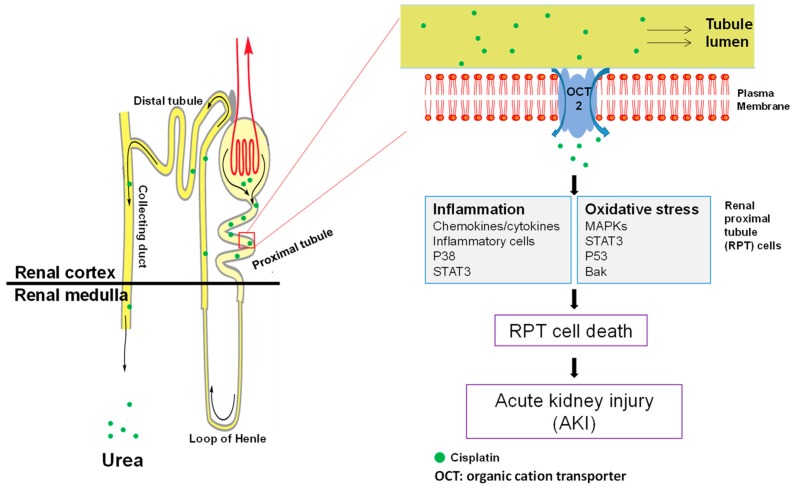
Disease pathophysiology of cisplatin nephrotoxicity. When passing through renal tubules, cisplatin is actively accumulated into RPT cells due to the abundance of OCT2 on the cell membrane of RPT cells. This leads to the massive production of intracellular ROS and inflammatory responses, both of which contribute to RPT cell death and subsequent acute kidney injury. RPT: renal proximal tubule; AKI: acute kidney injury; OCT: organic cation transporter.

After decades of studies, the pathophysiology underlying cisplatin nephrotoxicity has gradually been elicited. In this section, the possible signaling and molecular mechanisms underlying the disease pathophysiology are summarized and illustrated in Figure 3.

#### 3.3.1. Accumulation of Cisplatin in Kidney Cells

The clearance of cisplatin in mammals mainly occurs in the kidney by both glomerular filtration and tubular secretion [34]. In line with this, the concentration of cisplatin in the kidney largely exceeds its concentration in plasma, indicating an accumulation of the drug in renal cells [34]. This has been demonstrated with kidney slices and cultured renal proximal tubule segments [35,36]. In recent years, two different transporters, namely copper transporter 1 (Ctr1) and organic cation transporter 2 (OCT2), have been identified as responsible for the active transportation of cisplatin into mammalian cells. Ctr1 is highly expressed in both adult kidney and mammalian cells such as ovarian cancer cells [37]. Downregulation of Ctr1 in kidney cells attenuates cisplatin accumulation and subsequent toxicity [38], indicating that Ctr1 at least partially mediates the accumulation of cisplatin into kidney cells. However, whether Ctr1 plays a part in cisplatin-induced nephrotoxicity in vivo has not been studied. Unlike the universal expression of Ctr1, OCT2 is mainly located in renal proximal tubule cells [39]. Initially, it was found that cisplatin suppressed the transport of other OCT2 substrates into renal cells [39]. Conversely, an OCT2 substrate cimetidine decreases cisplatin uptake, and therefore cytotoxicity, in vitro [40], and also decreases nephrotoxicity in animals [33], suggesting a role of OCT2 in cisplatin-induced nephrotoxicity. This notion is strongly supported by a recent study which showed that OCT2 knockout mice exhibited higher resistance to cisplatin nephrotoxicity and lower urinary cisplatin excretion [32,33]. Consistently, certain mutations of the OCT2 gene are associated with reduced risk of cisplatin-induced nephrotoxicity in patients [32].

#### 3.3.2. Cell Death in Cisplatin-Induced Nephrotoxicity: Types and Location

Cisplatin-induced nephrotoxicity is characterized by massive tubular cell death, consisting of both necrosis and apoptosis [41]. Recent studies have suggested that dosage of cisplatin may determine whether cells undergo necrosis or apoptosis [42]. For example, millimolar concentrations of cisplatin cause tubular cell necrosis in hours, while lower concentrations of cisplatin (micromolar) lead to apoptosis in cultured tubular cells [42]. Nevertheless, it is also likely that the necrosis may be a result of apoptosis, termed secondary necrosis. It has been well recognized that renal tubules are the major site of cell death; however, it is not until recently that the type of cells injured by cisplatin were identified. By using proximal and distal tubule-specific lectins, it was observed that most apoptotic cells could be stained with a proximal tubule-binding lectin, namely phytohemagglutinin, while only a very small proportion of apoptotic cells were stained by peanut lectin agglutinin, which specifically stains distal tubule [43]. This result indicates that cisplatin mainly causes proximal tubule cell death, which may lead to subsequent renal dysfunction.

#### 3.3.3. Oxidative Stress in Cisplatin-Induced Nephrotoxicity

Oxidative stress has long been recognized as an important factor contributing to cisplatin nephrotoxicity [44]. Numerous studies have observed the massive production of reactive oxygen species (ROS) upon cisplatin treatment in cultured renal tubular cells, kidney slices, and in vivo animals [45,46]. Further studies have suggested three possible mechanisms that may account for the generation of ROS. First, cisplatin may cause mitochondrial ROS generation by suppressing the mitochondrial respiratory chain. For example, the activity of complex I-IV is reduced by 15–55% in 20 min after cisplatin treatment in tubule cells, which may result in ROS production [47]. However, one should bear in mind that ROS generation in this situation actually relies on residual electron flow through the mitochondrial respiratory chain; therefore, inhibition of the respiratory chain may block ROS accumulation [47]. Second, cisplatin may result in ROS production by activating NADPH oxidase. In agreement with this, pharmacological inhibition of NADPH oxidase protects renal cells in cultured proximal tubule cells and in vivo animals [48,49,50]. Finally, cisplatin may lead to ROS formation in microsomes via the cytochrome P450 (CYP) system. This is supported by evidence that knockout of CYP2E1 not only attenuates ROS accumulation but also alleviates cisplatin-induced renal injury [51]. In line with these findings, numerous antioxidants have been extensively studied in cisplatin nephrotoxicity and some of them are undergoing clinical trials [52].

#### 3.3.4. MAPK Activation in Cisplatin-Induced Nephrotoxicity

The MAPK (mitogen-activated protein kinase)-signaling pathways consist of the ERK, p38, and JNK pathways. The activation of MAPKs plays critical roles in the regulation of proliferation, differentiation, and apoptosis [53]. Emerging evidence suggests the activation of MAPKs in various experimental models of cisplatin-induced nephrotoxicity. For example, Nowak et al. first described that the activation of ERK led to its accumulation in mitochondria upon cisplatin treatment in primary renal tubule cells [54]. Pharmacological inhibition of ERK by PD98059 or U0126 largely ameliorates cisplatin-induced apoptosis [55]. In line with this, overexpression of constitutively active MEK1 leads to enhanced apoptosis, while dominant negative MEK1 decreases cisplatin-induced apoptosis in renal tubular cells. Importantly, ERK inhibitor U0126 attenuates in vivo cisplatin-induced nephrotoxicity [56], suggesting its involvement in cisplatin-induced renal toxicity. Similarly, p38 and JNK were also reported to be activated upon cisplatin treatment in animals [55]. Inhibition of either of the two kinases was reported to be protective in cisplatin-induced nephrotoxicity [57,58]. Besides, p38 may also take part in the regulation of TNFα expression and subsequent inflammatory response during cisplatin nephrotoxicity [57,58].

#### 3.3.5. Inflammation in Cisplatin-Induced Nephrotoxicity

Cisplatin-induced nephrotoxicity is associated with apparent inflammatory responses [59]. Massive expression of cytokines such as TNFα, IL-2, and MCP-1 has been reported in cisplatin-treated kidneys [60]. Deng et al. first showed that IL-10 can attenuate cisplatin-induced renal tissue injury and tubular apoptosis, suggesting that inflammatory response contributes to cisplatin nephrotoxicity [60]. Recent studies suggest that TNFα appears to be the key regulator of inflammatory response induced by cisplatin [60,61,62]. For example, TNFR2-deficient mice show higher resistance to cisplatin-induced nephrotoxicity compared with controls [61]. Moreover, Reeves and others showed that TNFα is crucial for the recruitment of inflammatory cells and upregulation of other proinflammatory factors because blockage of TNFα largely diminishes the effects mentioned above [62]. Recently, the initial production of TNFα during cisplatin nephrotoxicity was ascribed to proximal tubular cells instead of infiltrated inflammatory cells [63]. This further demonstrates the pivotal role of renal RPT cell in the pathogenesis of cisplatin-induced nephrotoxicity. Importantly, Faubel and colleagues showed that IL-1β, IL-18, and IL-6 may not significantly contribute to cisplatin-induced renal toxicity [64]. However, the involvement of other cytokines remains to be determined.

### 3.4. Prevention of Cisplatin-Induced Nephrotoxicity

Volume expansion by hydration has shown some success in the prevention of cisplatin nephrotoxicity [65]. However, the use of diuretics such as mannitol or furosemide fails to provide any beneficial effects in the scenario of cisplatin-induced nephrotoxicity in spite of their inclusion in many hydration regimens [66]. In fact, one comparative study observed aggravated nephrotoxicity in patients who received mannitol plus saline in comparison to those who received saline alone [23]. As a result, a recently issued clinical guideline suggests volume expansion with 0.9% saline and avoidance of diuretics [67]. Another approach aiming to prevent cisplatin-induced nephrotoxicity is to synthesize and screen novel analogues of cisplatin with lower toxicity to normal cells. In this direction, carboplatin, an analogue of cisplatin, has been approved for clinical usage of multiple cancers [68]. Compared with cisplatin, carboplatin presents less risk of gastrointestinal, renal and neurologic toxicity [68]; however, carboplatin appears to be less potent in terms of therapeutic effectiveness, at least in germ cell tumors, bladder cancers, and head and neck cancers, according to a clinical study [69].

## 4. Protective Effect of Hydrogen Sulfide in Cisplatin-Induced Nephrotoxicity

### 4.1. Role of Endogenous H_2_S in Cisplatin-Induced Nephrotoxicity

**Figure 4:**
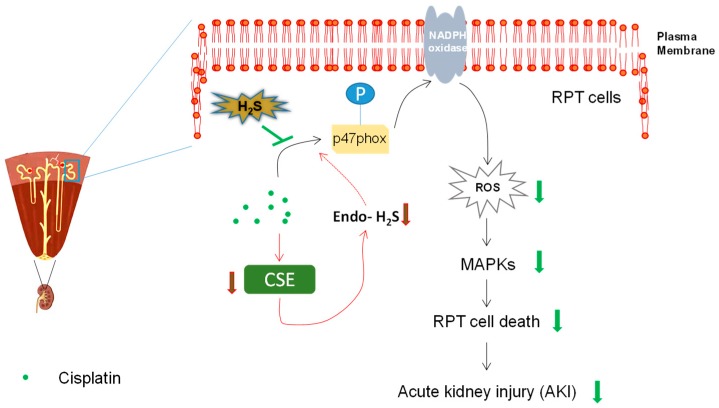
Protective effect of H_2_S in cisplatin-induced nephrotoxicity. Cisplatin led to the reduction of endogenous H_2_S by downregulating the expression of H_2_S-producing enzyme cystathionine γ-lyase (CSE), which may be involved in the subsequent renal proximal tubule (RPT) cell death and nephrotoxicity. Furthermore, H_2_S donors such as NaSH and GYY4137 ameliorated cisplatin-induced renal toxicity in vitro and in vivo, probably by suppression of NADPH oxidase activation, downstream reactive oxygen species (ROS) generation, and mitogen-activated protein kinases (MAPKs) activation.

The production of H_2_S is precisely controlled in the kidney by an enzymatic system. Nevertheless, changed expression levels of these H_2_S-producing enzymes were often found in various pathological conditions, which leads to altered levels of endogenous H_2_S [9,70]. Intriguingly, the alteration contributes to the progression of diseases such as renal ischemic–reperfusion injuries and diabetic nephropathy [9,70], perhaps due to significant roles of H_2_S not only in renal physiology but also in the maintenance of cellular homeostasis [14,71]. Although previous evidence suggests the reduced mRNA levels of CSE and CBS [72], whether and how this reduction influences the pathogenesis remains largely unexplored. We recently demonstrated the reduction of endogenous H_2_S levels due to the reduced protein level of CSE, but not CBS, upon cisplatin treatment [73]. Interestingly, antioxidant-like *N*-acetylcysteine (NAC) can almost completely abolish cisplatin-mediated CSE downregulation, consistent with a previous study showing that oxidative stress results in reduction of CSE [3]. Furthermore, the data also found that production of endogenous H_2_S is protective against cisplatin-induced RPT cell death, by overexpression of CSE or addition of H_2_S-producing substrates in RPT cells [73]. These results suggest that reduction of endogenous H_2_S may contribute to cisplatin-induced RPT cell injury (Figure 4). Nevertheless, further studies, such as examination of whether CSE deficiency worsens cisplatin-induced nephrotoxicity in animals, may be required to consolidate this conclusion. Moreover, how the reduction of endogenous H_2_S influences the disease progression may also need exploration in the future.

### 4.2. Donation of H_2_S Protects Against Cisplatin-Induced Nephrotoxicity

Although H_2_S donation exhibits broad protective effects in multiple organs, the therapeutic value of the strategy is controversial in the context of cisplatin-induced nephrotoxicity. For example, a study from Ahangarpour et al. showed that an H_2_S donor, NaSH, mitigated cisplatin-induced renal dysfunction and damage in rats [7]. In contrast to this study, a recent study reported that H_2_S slow releaser GYY4137 aggravated cisplatin-induced renal damage by increasing inflammatory response [72], which is controversial to the well-known role of H_2_S donors as anti-inflammatory substances [74,75,76,77]. However, several defects in the use of GYY4137 can be clearly identified in the study. For example, GYY4137 was prepared and stored in DMSO. This has been shown to be detrimental and accelerate GYY4137 decomposition [78]. Therefore, it is possible that the decomposed product of GYY4137 aggravated cisplatin-induced renal damage when dissolved in DMSO. Additionally, the study used a rather low dose of GYY4137 (21 mg/kg). It is likely that H_2_S might not be adequately produced considering the releasing property of H_2_S by the compound [79,80,81,82,83].

To resolve this disparity, we recently used several distinct H_2_S donors, namely H_2_S acute releaser NaSH, H_2_S slow releaser GYY4137, and H_2_S mitochondrial releaser AP39, in parallel to examine the effect of H_2_S in cisplatin-induced nephrotoxicity [73]. Our data indicated a strong protective effect of NaSH and GYY4137 in alleviating the cellular injuries caused by cisplatin treatment with a porcine-derived RPT cell line LLC-PK1. However, we did not observe any cytoprotective effect of AP39 (0.1–1 µM) in the same cell model [73]. Considering the main effect of AP39 in sequestering mitochondrial ROS [84,85], this could be explained by the lack of mitochondrial ROS generation in our cell model [73]. Actually, Takahashi et al. recently observed no mitochondrial ROS production in primarily cultured RPT cells [86], reflecting our results, which showed that NADPH oxidase might be the main ROS producer upon cisplatin treatment in RPT cells. The efficacy of NaSH and GYY4237 was later investigated in cisplatin-treated mice. A relatively high, yet commonly used, dose of GYY4137 (100 mg/kg; prepared in PBS) was used in the animal study [81,83]. Our data showed that H_2_S donors significantly mitigated cisplatin-induced increase of blood creatinine and urea nitrogen level, indicating the amelioration of renal function [73]. Consistently, the apoptotic level of renal cortex in mice receiving H_2_S donors but not vehicle was reduced. Taken together, the study may suggest a novel pharmaceutical usage for H_2_S in cisplatin-induced nephrotoxicity (Figure 4).

### 4.3. H_2_S Exhibited Anti-Oxidant Effect

Due to the role of ROS in the pathogenesis of cisplatin-induced nephrotoxicity [52], we mainly examined the antioxidant effects mediated by H_2_S. The results indicate that H_2_S significantly suppressed cisplatin-induced ROS generation by inhibiting the activation of NADPH oxidase. In fact, emerging evidence suggests that NADPH oxidase is a potential target of H_2_S [9]. Mechanistically, it seems that NaSH and GYY4137 led to significant S-sulfhydration of p47phox, indicating a direct interaction between H_2_S and p47phox. Similarly, it was shown that S-nitrosylation, mediated by NO and nitroxyl (a product of NO and H_2_S interaction), of crucial thiol residues on p47phox inhibits the activity of NADPH oxidase [87,88]. These results may imply a novel mechanism underlying H_2_S-mediated suppression of NADPH oxidase activity by directly S-sulfhydrating p47phox, although further studies are absolutely required to establish a definitive causality between the two events and to identify the cysteine residues targeted by H_2_S. Due to its suppressive effect of H_2_S on NADPH oxidase activation, it blunted the downstream ROS generation and MAPKs activation. Besides, the data also indicate the involvement of nucleus translocation of Nrf2 in H_2_S-mediated antioxidant effect [73]. This is not surprising, as a previous study demonstrated that H2S-mediated S-sulfhydration of Keap1 at cysteine-151 triggers the release and nucleus translocation of Nrf2 [89]. All of these may contribute to the protective effect of H_2_S in cisplatin-induced renal toxicity (Figure 2). In line with these findings, antioxidants have been extensively studied in cisplatin nephrotoxicity and some of them, such as *N*-acetylcysteine and silymarin, are undergoing clinical trials [42].

### 4.4. H_2_S Exhibited Anti-Apoptotic Effect

The most prominent feature of cisplatin-treated kidney is the apoptosis of cortical tissues, particularly in the region of RPT cells [42], which is obviously attenuated upon the treatment with H_2_S donors such as NaHS and GYY4137 in mice [73]. Consistently, cell apoptosis of cultured RPT cells is also mitigated upon cisplatin treatment when H_2_S is supplemented [73], indicating a strong antiapoptotic effect of H_2_S in cisplatin-induced RPT cell injury. In our cell model, we found that cisplatin-mediated activation of MAPKs contribute to subsequent RPT apoptosis because pharmacological inhibitors of MAPKs, particularly ERK and JNK, lessened cell apoptosis, while pretreatment with H_2_S donors largely prevented the phosphorylation of MAPKs [73]. This suggests that H_2_S-mediated antiapoptotic effect can be at least partially ascribed to its suppressive effect on MAPKs activation. Additionally, other antiapoptotic effects mediated by H_2_S may include prevention of cytochrome C release [90], activation of ATP potassium channels [75], etc.

As H_2_S exhibits pleiotropic activity in biology, it is highly likely that more potential mechanisms may underlie its therapeutic effect on cisplatin-induced nephrotoxicity. For example, H_2_S may inhibit inflammatory responses [74,77], which were shown to contribute cisplatin-induced renal injuries [59], and H_2_S has also been demonstrated to activate cell survival pathways that may counteract cisplatin-induced cytotoxicity [90,91]. These potential mechanisms in the context of cisplatin-induced nephrotoxicity have been summarized by Dugbartey et al. in a recent review paper [92].

### 4.5. Can H_2_S Enhance the Anti-Cancer Effect of Cisplatin?

The best approach to treat cisplatin-induced nephrotoxicity will probably be the one that can prevent renal dysfunction while at the same time enhance (or at least, not compromise) the anticancer activity of cisplatin. Intriguingly, H_2_S has been demonstrated, in recent years, as a novel anticancer molecule [93]. Therefore, it is conceivable to ask how H_2_S donors could influence the anticancer effect of H_2_S. It was noticed that NaSH, at the concentration that alleviated cisplatin-induced RPT cell injuries, did not affect the anticancer activity of cisplatin in cancer cell lines like MCF7 and HepG2 [73]. Interestingly, GYY4137 can add more anticancer activity to cisplatin, which is probably due to its anticancer effect, shown previously [94,95]. These data indicate a promising potential of H_2_S donors, particularly GYY4137, in prevention of cisplatin-induced renal toxicity. Nevertheless, whether H_2_S donors would affect the antitumor effect of cisplatin in tumor-bearing animals remains unknown.

## 5. Therapeutic Potential of Polysulfide in Cisplatin-Induced Nephrotoxicity

Polysulfides are a category of chemical compounds comprising chains of sulfur atoms. There are two main classes of polysulfide reported, namely anions and organic polysulfides. Anions have the general formula S_n_^2−^, which is the chemical basis of hydrogen polysulfide (H_2_S_n_) and sodium polysulfide (Na_2_S_n_). Organic polysulfides, such as garlic-derived diallyl disulfides (DADS) and diallyl trisulfides (DATS), usually possess a formula of RS_n_R, where R is either an alkyl or aryl group. Since anions such as sodium polysulfide (Na_2_S_n_) solely provide S_n_^2−^ in aqueous solutions, they should be able to more closely mimic endogenously produced polysulfide, and therefore are commonly used to explore the biological activity of polysulfide.

Similar to the effect of H_2_S, recent data showed that polysulfide also exhibited strong renal protective effects in cisplatin-induced nephrotoxicity by preventing renal dysfunction and apoptosis [96]. For example, it attenuates the membrane translocation of p47phox and therefore suppresses the activation of NADPH oxidase upon cisplatin treatment. Likewise, Akt activation was observed after addition of polysulfide, which results in the translocation of Nrf2 [96]. Remarkably, it was observed that that the number of sulfur atoms in polysulfide well reflects the efficacy of these molecules, not only in cell protection but also in cancer inhibition [96]. This may serve as a guide for further development of polysulfide donors for pharmaceutical usage. Nevertheless, the study only used the acute releaser of polysulfide, namely sodium polysulfide. Therefore, the development and study of more types of polysulfide donors might be warranted in the future.

Although evidence suggests that hydrogen polysulfide can be synthesized by H_2_S-producing enzyme 3-MST, the proportion of polysulfide derived from this pathway remains unclear. Moreover, the measurement of endogenous hydrogen polysulfide in biological samples is extremely difficult due to the abundance of other types of polysulfide such as organic polysulfide [15]. Therefore, the role of endogenous hydrogen polysulfide has not yet been explored. Nevertheless, the reduction of endogenous H_2_S implies that cisplatin may also reduce the level of endogenous hydrogen polysulfide as H_2_S at least partially serves as a source of hydrogen polysulfide in mammals [97,98]. This needs to be demonstrated in the future.

## 6. H_2_S and Polysulfide as A Remedy for Cisplatin-Mediated Toxicity in Other Organs?

Besides renal toxicity, administration of cisplatin often leads to other adverse effects such as ototoxicity, neutropenia, neurotoxicity, and liver toxicity. Interestingly, the disease mechanisms seem to be similar to that of nephrotoxicity (i.e., accumulation of cisplatin leads to excessive production of ROS and inflammatory responses which cause tissue damage). For example, recent studies demonstrated a critical role of NADPH oxidase in cisplatin-mediated neurotoxicity [99,100], which is very consistent with our findings in the renal system [73]. Thus, it is conceivable to speculate that H_2_S and polysulfide are likely to alleviate the toxicities caused by cisplatin in these organs. An examination of H_2_S and polysulfide effects on cisplatin-mediated antitumor activity and toxicities in different organs in the same tumor-bearing animals is definitely warranted, as it will provide us an answer as to whether H_2_S and polysulfide can serve as an effective combination therapy with cisplatin.

## 7. Future Perspectives and Conclusions

In spite many years’ effort, nephrotoxicity remains the main factor limiting the use of cisplatin in the treatment of cancer. Substances with anticancer activity probably serve as an ideal agent to treat cisplatin-induced nephrotoxicity as they may preserve and/or enhance the anticancer activity of cisplatin while limiting its toxic effects. Evidence is accumulated to suggest certain H_2_S donors are one such agent. However, there are key questions remaining to be addressed, such as (1) whether H_2_S donors can limit the renal toxicity but enhance the antitumor activity of cisplatin in tumor-bearing animals; (2) testing of drugs such as H_2_S donors, particularly those in clinical trials [93], as GYY4137 displays efficacy only at high concentration (in vitro: 400 µM and above; in vivo: 100 mg/kg); and (3) further mechanistic portrayal of H_2_S-mediated cytoprotective effects in RPT cells and inhibitory effects in cancer cells. The understanding of these issues will largely facilitate the translation of H_2_S into a novel therapy for cisplatin-induced nephrotoxicity.

## Abbreviations

H_2_S	Hydrogen sulfide
NO	Nitric oxide
CO	Carbon monoxide
CSE	Cystathionine γ-lyase
CBS	Cystathionine β-synthase
3-MST	3-mercaptopyruvate sulfurtransferase
CAT	Cysteine aminotransferase
DAO	d-amino acid oxidase
OCT2	Organic cation transporter 2
Ctrl1	Copper transporter 1
ROS	Reactive oxygen species
MAPK	Mitogen-activated protein kinase
NAC	*N*-Acetylcysteine
RPT	Renal proximal tubule
DADS	Diallyl disulfides
DATS	Diallyl trisulfides
